# Analysing time course microarray data using Bioconductor: a case study using yeast2 Affymetrix arrays

**DOI:** 10.1186/1756-0500-3-81

**Published:** 2010-03-19

**Authors:** Colin S Gillespie, Guiyuan Lei, Richard J Boys, Amanda Greenall, Darren J Wilkinson

**Affiliations:** 1 School of Mathematics & Statistics, Newcastle University, Newcastle upon Tyne, NE1 7RU, UK; 2 Centre for Integrated Systems Biology of Ageing and Nutrition (CISBAN), Newcastle University, UK; 3 Institute for Ageing and Health, Newcastle University, Campus for Ageing and Vitality, Newcastle upon Tyne, NE4 5PL, UK

## Abstract

**Background:**

Large scale microarray experiments are becoming increasingly routine, particularly those which track a number of different cell lines through time. This time-course information provides valuable insight into the dynamic mechanisms underlying the biological processes being observed. However, proper statistical analysis of time-course data requires the use of more sophisticated tools and complex statistical models.

**Findings:**

Using the open source CRAN and Bioconductor repositories for R, we provide example analysis and protocol which illustrate a variety of methods that can be used to analyse time-course microarray data. In particular, we highlight how to construct appropriate contrasts to detect differentially expressed genes and how to generate plausible pathways from the data. A maintained version of the R commands can be found at http://www.mas.ncl.ac.uk/~ncsg3/microarray/.

**Conclusions:**

CRAN and Bioconductor are stable repositories that provide a wide variety of appropriate statistical tools to analyse time course microarray data.

## Introduction

As experimental costs decrease, large scale microarray experiments are becoming increasingly routine, particularly those which track a number of different cell lines through time. This is because time-course information provides valuable insight into the dynamic mechanisms underlying the biological processes being observed. However, a proper statistical analysis of time-course data requires the use of more sophisticated tools and complex statistical models. For example, problems due to multiple comparisons are increased by catering for changing effects over time. In this case study, we demonstrate how to analyse time-course microarray data by investigating a data set on yeast. We discuss issues related to normalisation, extraction of probesets for specific species, chip quality and differential expression. We also discuss network inference in the Additional file 1. The freely available software system R (see [1,2]) has many benefits for analysing data of this type and so throughout the analysis we give the R commands that produce the numerical/graphical output shown in this paper. A maintained version of the R commands can be found at http://www.mas.ncl.ac.uk/~ncsg3/microarray/.

### Description of the data

The data were collected according to the experimental protocol described in [3]. Briefly, three biological replicates were studied on each of a wild-type (WT) yeast strain and a strain carrying the *cdc13-1 *temperature sensitive mutation (in which telomere uncapping is induced by growth at temperatures above around 27°C). These replicates were sampled initially at 23°C (at which *cdc13-1 *has essentially WT telomeres) and then at 1, 2, 3 and 4 hours after a shift to 30°C to induce telomere uncapping. The thirty resulting RNA samples were hybridised to Affymetrix yeast2 arrays. The microarray data are available in the ArrayExpress database (see [4]) under accession number E-MEXP-1551 .

## Loading microarray data into Bioconductor

### Installing Bioconductor and associated packages

Assuming that R is already installed, Bioconductor is fairly straightforward to obtain installation script, viz:

> **url**='http://bioconductor.org/biocLite.R'

> **source**(**url**)

> biocLite()

This installs a number of base packages, including affy, affyPLM, limma, and gcrma (see [5-7]). Additional non-standard packages can also be easily installed. For example, the additional packages needed for this paper can be installed by using

> *#From Bioconductor*

> biocLite(**c**('ArrayExpress', 'Mfuzz', 'timecourse', 'yeast2.db', 'yeast2probe'))

> *#From cran*

> **install.packages**(**c**('GeneNet', 'gplots'))

Bioconductor packages are updated regularly on the web and so users can easily update their currently installed packages by starting a new R session and then using

> **update.packages**(repos = biocinstallRepos())

See [8] for further details on installation.

A list of packages used in this paper is given in the Additional file 1.

### Entering data into Bioconductor

The data used in this paper can be downloaded from ArrayExpress into R using the commands

> **library**(ArrayExpress)

> yeast.raw = ArrayExpress('E-MEXP-1551')

Unfortunately due to changes in the ArrayExpress website, the ArrayExpress package for Bioconductor 2.4 (the default version for R 2.9) produces an error and so we must use the package in Bioconductor 2.5 (the default version for R 2.10). Details for downloading the latest ArrayExpress package can be found in the Additional file 1.

A brief description of the yeast.raw object can be obtained by using the print(yeast.raw) command:

AffyBatch object

size of arrays = 496 × 496 features (3163 kb)

cdf = Yeast_2 (10928 affyids)

number of samples = 30

number of genes = 10928

annotation = yeast2

If the Affymetrix microarray data sets have been downloaded into a single directory, then the .cel files can be loaded into R using the ReadAffy command.

Also available from ArrayExpress are the experimental conditions. However, some preprocessing is necessary:

> ph = yeast.raw@phenoData

> exp_fac = **data.frame**(data_order = **seq **(1, 30),

+                                     strain = ph@data**$**Factor.Value.GENOTYPE.,

+                                     replicates = ph@data**$**Factor.Value.INDIVIDUAL.,

+                                     tps = ph@data**$**Factor.Value.TIME.)

> **levels**(exp_fac**$**strain) = **c**('m', 'w')

> exp_fac = **with**(exp_fac, exp_fac[**order**(strain, replicates, tps), ])

> exp_fac**$**replicate = **rep**(**c**(1, 2, 3), each = 5, 2)

The data frame exp_fac stores all the necessary information, such as strain, time and replicate, which are necessary for the statistical analysis.

Note that there are two yeast species on this chip, *S. pombe *and *S. cerevisiae*. Also, amongst the 10,928 probesets (with each probeset having 11 probe pairs), there are 5,900 *S. cerevisiae *probesets.

## Pre-processing

### Extraction of *S. cerevisiae *probesets

As these microarrays contain probesets for both *S. cerevisiae *and *S. pombe*, we first need to extract the *S. cerevisiae *data before normalisation. This can be done by filtering out the *S. pombe *data using the s_cerevisiae.msk file from the Affymetrix website (see [9]). Note that users first need to register with the Affymetrix website before downloading this file. Also note that in our analysis, the transcript id i.e. the systematic orf name (obtained from [10]) is used for genes with no name.

We obtain a data frame containing lists of *S. cerevisiae *genes, probes and transcripts (using the function ExtractIDs() in the Additional file 1) as follows

> *#Read in the mask file*

> s_cer = **read.table**('s_cerevisiae.msk', skip = 2, stringsAsFactors = FALSE)

> probe_filter = s_cer[[1]]

> **source**('ExtractIDs.R')

> c_df = ExtractIDs(probe_filter)

We also need to restrict the view of yeast.raw to the *x*- and *y*- coordinates of the *S. cerevisiae *probesets in the cdf environment by using

> *#Get the raw dataset for S. cerevisiae only*

> **library**(affy)

> **library**(yeast2probe)

> **source**('RemoveProbes.R')

> cleancdf = cleancdfname(yeast.raw@cdfName)

> RemoveProbes(probe_filter, cleancdf, 'yeast2probe')

Note that the commands in RemoveProbes.R are listed in the Additional file 1. Thus the attributes of yeast.raw, obtained via print(yeast.raw), are now

AffyBatch object

size of arrays = 496 × 496 features(3167 kb)

cdf = Yeast_2(5900 affyids)

number of samples = 30

number of genes = 5900

annotation = yeast2

and the number of genes (actually probesets here) is 5,900 now that the *S. pombe *probesets have been removed.

### Data Quality Assessment

Before any formal statistical analysis, it is important to check for data quality. Initially, we might examine the perfect and mismatch probe-level data to detect anomalies. Images of the first five arrays can be obtained using

> op = **par**(mfrow = **c**(3, 2))

> **for**(iin 1:5) {

+          plot_title = **paste **('Strain:', exp_fac**$**strain [i], 'Time:', exp_fac**$**tps [i])

+          d = exp_fac**$**data_order [i]

+          **image**(yeast.raw [, d], main = plot_title)

+ }

These commands produce the image shown in the Additional file 1: Figure S2. Data quality can be assessed by examining such images for anything that appears non-random such as rings, shadows, lines and strong variations in shade. The images for our data set do not appear to have any non-random structure and so data quality is probably high.

Another useful quality assessment tool is to examine density plots of the probe intensities. The command

> d = exp_fac**$**data_order[1:5]

> **hist**(yeast.raw[, d], lwd = 2, ylab = 'Density', xlab = 'Log (base 2) intensities')

produces the image shown in the Additional file 1: Figure S3. Typically, differences in spread and position are corrected by normalisation. However, the appearance of significant multi-modality in the distribution or many outlying observations are indicative of poor data quality.

Other exploratory data analysis techniques that should be carried include MAplots, where two microarrays are compared and their log intensity difference for each probe on each gene are plotted against their average. Also of interest is to examine RNA degradation (see [6]), although [11] cast some doubt over the validity of this method. For details on how to carry out both of these methods in R, see [12,13] for detailed instructions.

### Normalising Microarray Data

There are number of methods for normalising microarray data. Two of the most popular methods are GeneChip RMA (GCRMA) and Robust Multiple-array Average (RMA); see [14,15]. Essentially, GCRMA and RMA differ in how they deal with background noise, with GCRMA using a more sophisticated correction algorithm. However, the approach adopted by GCRMA means that it can be time-consuming to use with large data sets in contrast to RMA. A potential drawback of using RMA is that it assumes that the overall levels of expression are similar for each array. However this assumption may be invalid if, for example, mutant cells have a radically different level of transcriptional activity than the WT. For further information regarding normalising microarray data sets, see for example [16,17].

Since we have thirty microarray data sets and believe that the levels of transcriptional activity are similar across strains, we will use the RMA normalisation method. This technique normalises across the set of hybridizations at the probe level. The data can be normalised via

> yeast.rma = rma(yeast.raw)

> yeast.matrix = exprs(yeast.rma)[, exp_fac**$**data_order]

> cnames = **paste**(exp_fac**$**strain, exp_fac**$**tps, sep = ' ')

> **colnames**(yeast.matrix) = cnames

> exp_fac**$**data_order = 1:30

The normalisation procedure consists of three steps: model-based background correction, quantile normalisation and robust averaging. The aim of the quantile normalisation is to make the distribution of probe intensities for each array in a set of arrays the same. We illustrate its effect by studying boxplots of the raw *S. cerevisiae *data against their normalised counterparts values, shown in the Additional file 1: Figure S4. Boxplots provide a useful graphical view of data distributions and contain their median, quartiles, maximum and minimum values. The boxplot command is in the affyPLM package and so the figure is produced by using

> **library**(affyPLM)

> **par**(mfrow = **c**(1, 2))

> *#Raw data intensities*

> **boxplot**(yeast.raw, **col **= 'red', main="")

> *#Normalised intensities*

> **boxplot**(yeast.rma, **col **=**'**blue')

### Principal Component Analysis

Principal component analysis (PCA) is useful in exploratory data analysis as it can reduce the number of variables to consider whilst still retaining much of the variability in the data. In particular, PCA is useful for identifying patterns in the data. Essentially, principal components partition the data into orthogonal linear components which explain different contributions to the variability in the data. The first component explains the largest contribution to variability in the original dataset, that is, retains most information, with the second component explaining the next largest contribution to variability, and so on. The following commands calculate the principal components

> yeast.PC = **prcomp**(**t**(yeast.matrix))

> yeast.scores = **predict**(yeast.PC)

which we can then plot using

> *#Plot of the first two principal components*

> **plot**(yeast.scores [, 1], yeast.scores [, 2],

+             xlab = 'PC 1', ylab = 'PC 2',

+             pch = **rep**(**seq **(1, 5), 6),

+             **col **= **as.numeric**(exp_fac**$**strain))

> **legend**(-20, -4, pch = 1:5, cex = 0.6, **c**('t 0', 't 60', 't 120', 't 180', 't 240'))

**Figure 1:**
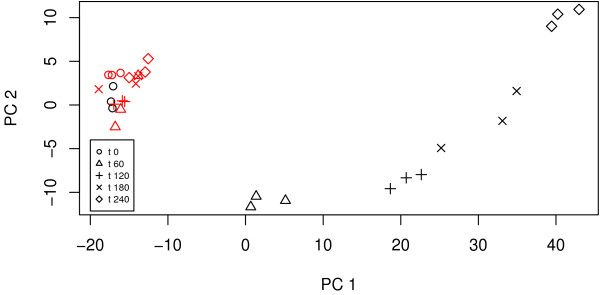
**A plot of the first two principal components**. The red symbols correspond to the wild-type strain.

Figure 1 highlights a clear (and expected) time effect in the mutant yeast which is not present in the wild-type strain. In particular, mutant samples are clustered by their time points; for example, the three mutant replicates at time point 4 are clustered at the bottom right of the figure.

## Identifying differentially expressed genes

In this experiment, interest lies in differences in gene expression over time between the wild-type and mutant yeast strains. It is expected that the wild-type expression level is independent of time. Also we anticipate that the mutant expressions at time *t *= 0 are the same as the wild-type expression level. This hypothesis is supported by the PCA plot in Figure 1.

There are currently two main packages available to detect differentially expressed genes using this kind of data: the timecourse package and the limma package. We illustrate how to analyse these data using both packages.

### Using the timecourse package

This package assesses treatment differences by comparing time-course mean profiles allowing for variability both within and between time points. It uses the multivariate empirical Bayes model proposed by [18].

Further details of the timecourse package can be found in [19]. After installing the timecourse library, we construct a size matrix describing the replication structure using

> **library**(timecourse)

> size = **matrix**(3, **nrow **= 5900, **ncol **= 2)

To extract a list of differentially expressed we calculate the Hotelling statistic  via

> c.grp = **as.character**(exp_fac**$**strain)

> t.grp = **as.numeric**(exp_fac**$**tps)

> r.grp = **as.character**(exp_fac**$**replicate)

> MB.2D = mb.long(yeast.matrix, times = 5, method = '2', reps = size,

+                               condition.grp = c.grp, time.grp = t.grp, rep.grp = r.grp)

The top (say) one hundred genes can be extracted via

> gene_positions = MB.2D**$**pos.HotellingT2 [1:100]

> gnames = **rownames**(yeast.matrix)

> gene_probes = gnames[gene_positions]

The expression profiles can also be easily obtained. The profile for the top ranked expression is found using

> plotProfile(MB.2D, ranking = 1, gnames = **rownames**(yeast.matrix))

and is shown in the Additional file 1: Figure S5.

### Using the limma package

The limma package uses the moderated *t*-statistic described by [7,20]. The function lmFit within the limma library fits a linear model for each gene for a given series of arrays, where the coefficients of the fitted models describe the differences between the RNA sources hybridised to the arrays. Precisely, we fit the model *E *[*y*_*g*_] = *Xα *_*g*_, where *y*_*g *_= (*y*_*g*,1_, ..., *y*_*g, n*_)^*T *^contains the expression values for gene *g *across the *n *arrays, *X *is a design matrix which describes key features of the experimental design used and *α*_*g *_is the coefficient vector for gene *g*. In the analysis studied here, the yeast data consists of data from *n *= 30 arrays. The entries in the columns of *X *depend on the experimental design used: there are two yeast strains (mutant and wild type), each measured at five separate time points, and we are interested in comparing the gene expressions between mutant and wild type strains over time. Thus we seek a linear model describing the ten strain × time combinations by determining values for the ten coefficients in the coefficient vector *α*_*g*_. We will label these ten coefficients as ('m0', 'm60, 'm120', 'm180', 'm240', 'w0', 'w60', 'w120', 'w180', 'w240'), where the first five coefficients represent the levels of the mutant strain at time points *t *= 0, 1, 2, 3, 4 and the remaining five coefficients are the equivalent versions for the wild type strain. Statistically speaking, the model has a single factor with ten levels. The design matrix *X *links these factors to the data in the arrays by having zero entries except when an array contributes an observation to a particular strain × time combination. For example, array 26 measures the expression of the first wild type microarray at time *t *= 0 and so contributes an observation to level 'w0', the sixth strain × time combination. Thus the entry in row 26, column 6 of the design matrix *X*(26, 6) = 1. Further, the arrays are arranged in groups of three replicates. Thus the overall experimental structure (expt_structure below) has three arrays on level 'm0', then three arrays on 'm60', and so on. Setting up the factor levels and the design matrix is done in R by using

> **library**(limma)

> expt_structure = **factor**(**colnames**(yeast.matrix))

> *#Construct the design matrix*

> X = **model.matrix**(~0 + expt_structure)

> **colnames**(X) = **c**('m0', 'm60', 'm120', 'm180', 'm240', 'w0', 'w60', 'w120', 'w180', 'w240')

and then the coefficient vector *α*_*g *_is estimated via the command

> **lm.fit **= lmFit(yeast.matrix, X)

Determining the differentially expressed genes amounts to studying contrasts of the various strain × time levels, as described by a contrast matrix *C*. For these data, we are mainly interested in differences at the later time points, and so a possible set of contrasts to investigate is that of differences between the mutant and wild type strains at each time point, that is, ('m60-w60', 'm120-w120', 'm180-w180', 'm240-w240'). The limma package allows complete flexibility over the choice of contrasts, however this necessarily includes an additional level of complexity. The values in the coefficient vector of contrasts, *β*_*g *_= *C*^*T*^*α*_*g *_for gene *g*, describe the size of the difference between strains at each time point. The relevant R commands are

> mc = makeContrasts('m60-w60', 'm120-w120', 'm180-w180', 'm240-w240', **levels **= X)

> c.fit = contrasts.fit(**lm.fit**, mc)

> eb = eBayes(c.fit)

The final command uses the eBayes function to produce moderated *t*-statistics which assess whether individual contrast values *β*_*gj *_are plausibly zero, corresponding to no signifficant evidence of a difference between strains at time point *j*. The moderated *t*-statistic is constructed using a shrinkage approach and so is not as sensitive as the standard *t*-statistic to small sample sizes. It also gives a moderated *F*-statistic which can be used to test whether all contrasts are zero simultaneously, that is, whether there is no difference between strains at all time points.

### Ranking differentially expressed genes

There are a number of ways to rank the differentially expressed genes. For example, they can be ranked according to their log-fold change

> *#see help(toptable) for more options*

> toptable(eb, sort.by = 'logFC')

or by using *F*-statistics

> topTableF(eb)

The advantage of using *F*-statistics over the log fold change is that the *F*-statistic takes into account variability and reproducibility, in addition to fold-change.

Our analysis is based on a large number of statistical tests, and so we must correct for this multiple testing. In our example we use the (very) conservative Bonferroni correction since we have a large number of differentially expressed genes and the resulting corrected list is still long. Another common method of correcting for multiple testing is to use the false discovery rate (fdr) (use the command ?p.adjust to obtain further details). The following commands rank genes according to their (corrected) *F*-statistic *p*-value and annotates the output by indicating the direction of the change for each contrast for each gene: +1 for up-regulated expression (mutant type having higher expression than wild type at a particular time point), -1 for down-regulated expression and 0 for no significant change.

> modFpvalue = eb**$**F.p.value

> *#Change 'bonferroni' to 'fdr' to use the false discovery rate as a cut*-*off*

> indx = **p.adjust**(modFpvalue, method = 'bonferroni') < 0.05

> sig = modFpvalue[indx]

> *#No. of sig. differential expressed genes*

> nsiggenes = **length**(sig)

> results = decideTests(eb, method = 'nestedF')

> modF = eb**$F**

> modFordered = **order**(modF, decreasing = TRUE)

> *#Retrieve the significant probes and genes*

> c_rank_probe = c_df**$**probe [modFordered [1:nsiggenes]]

> c_rank_genename = c_df**$**genename [modFordered [1: nsiggenes]]

> *#Create a list and write to a file*

> updown = results[modFordered [1:nsiggenes],]

> **write.table**(**cbind**(c_rank_probe, c_rank_genename, updown),

+                      **file **= 'updown.csv', sep = ',', **row.names **= FALSE, **col.names **= FALSE)

**Figure 2:**
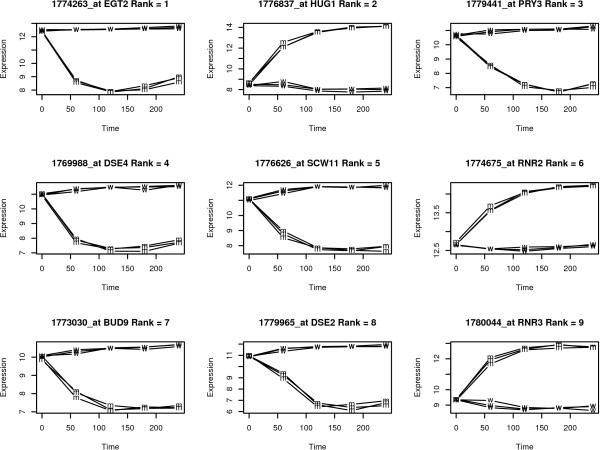
**Time course expression levels for the top 9 differentially expressed genes, ranked by their *F*-statistic**.

The following code (adapted from lecture material found at [13]) plots the time course expression for the top one hundred differentially expressed genes according to their *F*-statistic (see Figure 2).

> *#Rank of Probesets, also output gene names*

> **par**(mfrow = **c **(3, 3), ask = TRUE, cex = 0.5)

> **for **(i in 0:99){

+    indx = **rank**(modF) == **nrow**(yeast.matrix) -i

+    

+    id = c_df**$**probe [indx]

+    name = c_df**$**genename [indx]

+    genetitle = **paste**(**sprintf **('%. 30s', id), **sprintf **('%. 30s', name), 'Rank =', i +1)

+    

+    exprs.row = yeast.matrix[indx, ]

+    

+    **plot **(0, pch = NA, xlim = **range**(0, 240), ylim = **range**(exprs.row), ylab = 'Expression',

+                   xlab = Time, main = genetitle)

+    

+    **for **(j in 1:6){

+       pch_value = **as.character**(exp_fac**$**strain [5 * j])

+       **points**(**c **(0, 60, 120, 180, 240), exprs.row[(5 * j-4):(5 * j)], type = 'b', pch = pch_value)

+    }

+ }

When interpreting rank orderings based on statistical significance, it is important to bear in mind that a statistically significant differential expression is not always biologically meaningful. For example, Figure 2 contains *RNR2*. This gene is highly significant because of low variation in its time course. However the actual difference in expression levels between wild-type and mutant stains is relatively small. We address this problem in the next section.

### Comparison of the timecourse and limma packages

Both packages have different strengths. One advantage of the timecourse package over the limma package is that it allows for correlation between repeated measurements on the same experimental unit, thereby reducing false positives and false negatives; these false positives/negatives are a significant problem when the variance-covariance matrix is poorly estimated. An advantage of the limma package is that it allows more flexibility by allowing users to construct different contrasts. In general we might expect both packages to produce fairly similar lists of say the top 100 probesets. In the analysis of the yeast data, we can determine the overlap of the top 100 probesets by using

> N = 100

> gene_positions = MB.2D**$**pos.HotellingT2[1:N]

> tc_top_probes = gnames[gene_positions]

> lm_top_probes = c_df**$**probe[modFordered[1:N]]

> **length**(**intersect**(tc_top_probes, lm_top_probes))

The result is a moderately large overlap of fifty-three probesets. We note that changing the ranking method in the limma package also yields similar results as those given by the timecourse library.

### Two fold-change list

When looking for "interesting" genes it can be helpful to restrict attention to those differential expressed that are both statistically significant and of biological interest. This objective can be achieved by considering only significant genes which show, say, at least a two-fold change in their expression level. This gene list is obtained using the following code (adapted from [12])

> *#Obtain the maximum fold change but keep the sign*

> maxfoldchange = **function**(foldchange)

+    foldchange[**which.max**(**abs**(foldchange))]

> difference = **apply**(eb**$**coeff, 1, maxfoldchange)

> pvalue = eb**$**F.p.value

> lodd = -log10(pvalue)

> *#hfc: high fold*-*change*

> nd = (**abs**(difference) > **log **(2, 2))

> ordered_hfc = **order**(**abs**(difference), decreasing = TRUE)

> hfc = ordered_hfc[1: **length**(difference[nd])]

> np = **p.adjust**(pvalue, method = ' bonferroni') < 0.05

> *#lpv: low p value(large F*-*value)*

> ordered_lpv = **order**(**abs**(pvalue), decreasing = FALSE)

> lpv = ordered_lpv[1: **length **(pvalue[np])]

> oo = **union**(lpv, hfc)

> i i = **intersect**(lpv, hfc)

**Figure 3:**
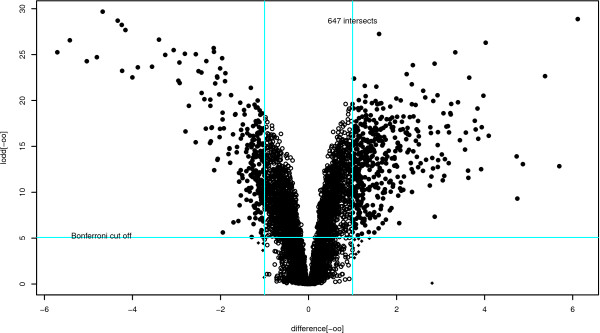
**Volcano plot showing the Bonferroni cut-off and the two-fold change**.

Figure 3 contains a "volcano" plot which illustrates the effect of using different levels of fold change and significance thresholds. The figure is produced by using the following code

> *#Construct a volcano plot using moderated F-statistics*

> **par**(cex = 0.5)

> **plot**(difference[-oo], lodd[-oo], xlim = **range**(difference), ylim = **range**(lodd))

> **points**(difference[hfc], lodd[hfc], pch = 18)

> **points**(difference[lpv], lodd[lpv], pch = 1)

> *#Add the cut *- *off lines*

> **abline**(v = **log **(2, 2), **col **= 5); **abline**(v = - log (2, 2), **col **= 5)

> **abline **(h = -log10 (0.05**/**5900), **col **= 5)

> **text**(**min**(difference) + 1, -log10 (0.05**/**5900) + 0.2, 'Bonferroni cut off')

> **text**(1, **max**(lodd) - 1, **paste **(**length **(i i), 'intersects'))

## Cluster Analysis

Biological insight can be gained by determining groups of differentially expressed genes, that is, groups of genes which increase or decrease simultaneously. This can be achieved by using cluster analysis.

### Traditional cluster analysis

In this section, we separate the top fifty differentially expressed genes into groups of similar pattern (clusters). Clearly different genes will have different overall levels of expression and so we first standardise their measurements by taking the expression level of the mutant strain (at each time point) relative to the wild-type at time *t *= 0:

> c_probe_data = yeast.matrix [ii,]

> *#Average of WT*

> wt_means = **apply**(c_probe_data [, 16:30], 1, **mean**)

> m = **matrix**(**nrow **= **dim**(c_probe_data) [1], **ncol **= 5)

> **for **(i in 1:5) {

+       mut_rep = **c**(i, i+5, i +10)

+       m [, i] = **apply**(c_probe_data [, mut_rep], 1, **mean**) - wt_means

+ }

> **colnames**(m) = **sort**(**unique**(exp_fac**$**tps))

**Figure 4:**
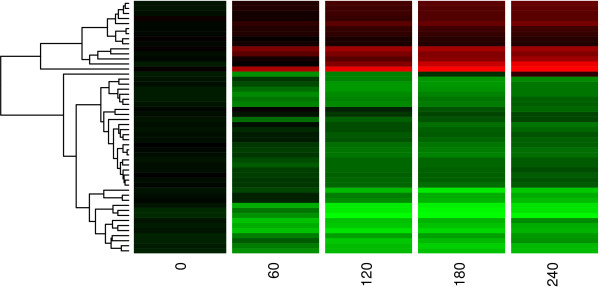
**Clustering of the top fifty differentially expressed genes**. Red and green correspond to up- and down-regulation respectively.

The heatmap in Figure 4 is obtained by using the function heatmap.2 from the library gplots via the following code

> **library**(gplots)

> *#Cluster the top 50 genes*

> heatmap.2 (m [1:50,], dendrogram = 'row', Colv = FALSE, **col **= greenred (75),

+                   key = FALSE, keysize = 1.0, symkey = FALSE, density.info = ' none',

+                   **trace **= 'none', colsep = **rep**(1:10), sepcolor = 'white', sepwidth = 0.05,

+                   hclustfun = **function **(**c**){**hclust**(**c**, method = 'average')},

+                   labRow = NA, cexCol = 1)

Figure 4 shows the relative expression levels for the mutant strain at each time point ('0', '60', '120', '180', '240'). As expected, the relative expression levels at time *t *= 0 are very similar. However, as time progresses, groupings of genes appear whose levels are up-regulated (red) or down-regulated (green). Note that the intensity of the colour corresponds to the magnitude of the relative expression. Gene names appear on the right side of the figure and on the left side, the cluster dendrogram shows which genes have similar expression. The dendrogram suggests that there are perhaps six to ten clusters.

### Soft clustering

Soft clustering methods have the advantage that a probe can be assigned to more than one cluster. Furthermore, it is possible to grade cluster membership within particular groupings. Soft clustering is considered more robust when dealing with noisy data; for more details see [21,22]. The Mfuzz package implements soft clustering using a fuzzy c-means algorithm. Analysing the data for *c *= 8 clusters is achieved by using

> **library**(Mfuzz)

> tmp_expr = **new**('ExpressionSet', exprs = m)

> cl = mfuzz(tmp_expr, **c **= 8, m = 1.25)

> mfuzz.plot(tmp_expr, cl = cl, mfrow = **c**(2, 4), new.window = FALSE)

**Figure 5:**
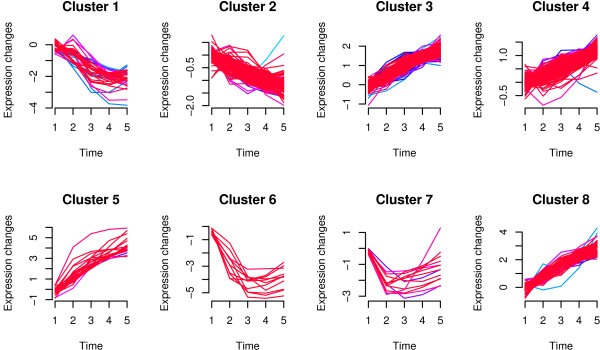
**Eight clusters obtained using the **Mfuzz** package**.

Of course, it is usually not clear how many clusters there are (or should be) within a dataset and so the sensitivity of conclusions to the choice of number of clusters (*c*) should always be investigated. For example, if *c *is chosen to be too large then some clusters will appear sparse and this might suggest choosing a smaller value of *c*. Figure 5 shows the profiles of the eight clusters obtained from the Mfuzz package. The probes present within each cluster can be found by using

> cluster = 1

> cl [[4]][,cluster]

## Conclusion

The response to telomere uncapping in *cdc13-1 *strains was expected to share features in common with responses to cell cycle progression, environmental stress, DNA damage and other types of telomere damage. The statistical analysis determined lists of probesets associated with genes involved in all of these processes. The techniques used focussed on making best use of the temporal information in time-course data. The use of *cdc13-1 *strains, which uncap telomeres quickly and synchronously, also allowed the identification of genes involved in the acute response to telomere damage. This case study has demonstrated the power of R/Bioconductor to analyse time-course microarray data. Whilst the statistical analysis of such data is still an active research area, this paper presents some of the cutting-edge tools that are available to the life science community. All software discussed in this article is free, with many of the packages being open-source and subject to on-going development.

## Competing interests

The authors declare that they have no competing interests.

## Authors' contributions

AG conducted the microarray experiments. All authors participated in the analysis of the data and in the writing of the manuscript.

## Supplementary Material

Additional file 1 **Additional R commands and analysis**. 1. R commands for extracting S. cerevisiae ids, removing unwanted probesets and converting probesets to genes. 2. R commands for genetic regulatory network inference. 3. A list of R packages used in this manuscript. 4. Additional figures. Click here for file
