# Supplementary material for: Analysing time course microarray data using Bioconductor: a case study using yeast2 Affymetrix arrays
Source: BMC Res Notes. 2010 Mar 19;3:81. doi: 10.1186/1756-0500-3-81 (PMC2880961; doi:10.1186/1756-0500-3-81)
Supplement: Additional file 1 — Additional R commands and analysis. 1. R commands for extracting S. cerevisiae ids, removing unwanted probesets and converting probesets to genes. 2. R commands for genetic regulatory network inference. 3. A list of R packages used in this manuscript. 4. Additional figures. [file 1756-0500-3-81-S1.PDF]

Additional File 1

1 R commands for extracting S. cerevisiae ids, removing unwanted probesets and converting probesets to genes

R function for extracting S. cerevisiae ids

As these microarrays contain probesets for both *S. cerevisiae* and *S. pombe*, we first need to extract the *S. cerevisiae* data before normalisation. This can be done by filtering out the *S. pombe* data using the `s_cerevisiae.msk` file from the Affymetrix website (see [1]). Note that the transcript id, i.e. the systematic orf name (obtained from [2]) will be used for genes with no name.

```
> #probe_filter: a vector of S. cerevisiae genes
> ExtractIDs = function(probe_filter)
+ {
+     #Get both S. pombe & S. cerevisiae ids from yeast2GENENAME library
+     library(yeast2.db)
+     genenames = as.list(yeast2GENENAME)
+     probes = names(genenames)
+
+     #Get all transcript ids from yeast2annotation.csv
+     annotations = read.csv(file='yeast2annotation.csv', header=TRUE,
+                            stringsAsFactors=FALSE)
+     transcript_id = annotations[,3]
+     probeset_id = annotations[,1]
+
+     #Reorder the transcript_id to match probes
+     transcript_id = transcript_id[match(probes, probeset_id)]
+
+     #Retrieve the probeset and transcript ids for S. cerevisiae
+     c_probe_id = probes[-match(probe_filter, probes)]
+     c_transcript_id = transcript_id[-match(probe_filter, probes)]
+
+     #We need the TranscriptID if the gene name is `NA'
+     yeast_genenames = transcript_id
+     for(i in seq(along=probeset_id))
+     {
+         gname = genenames[i][[1]]
+         if(!is.na(gname))
+             yeast_genenames[i] = gname
+     }
+
+     #Set the gene name
+     c_genename = yeast_genenames[-match(probe_filter, probes)]
+     df = data.frame(probe=c_probe_id, transcript=c_transcript_id, genename=c_genename,
+                     stringsAsFactors=FALSE)
+     return(df)
+ }
```

R function for removing unwanted probesets

If an Affymetrix microarray chip contains more than one species then it can be useful to focus on a particular species and filter out the unwanted probesets. For example, the `yeast2.db` Affymetrix chip contains both *S. pombe* and *S. cerevisiae* yeast species. To filter out the *S. pombe* probesets, we remove the

mappings from the $x-$, $y-$coordinates to the *S. pombe* probesets in the cdf environment. The following function (adapted from [3]) removes the unwanted *S. pombe* instances:

```
> #listOutProbeSets: Probes sets that are removed.
> #cdfpackagename: The cdf package name.
> #probepackagename: The probe package name.
> RemoveProbes=function(listOutProbeSets, cdfpackagename, probepackagename){
+
+     require(cdfpackagename, character.only=TRUE)
+     require(probepackagename, character.only=TRUE)
+
+     #Default probe dataset values
+     probe.env.orig = get(probepackagename)
+
+     #Remove probesets from the CDF environment
+     if(!is.null(listOutProbeSets))
+         rm(list=listOutProbeSets, envir=get(cdfpackagename))
+
+     #Set the PROBE env accordingly (idea originally from gcrma compute.affinities.R)
+     tmp = get('xy2indices', paste('package:', cdfpackagename, sep=''))
+
+     newAB    = new('AffyBatch', cdfName=cleancdf)
+     pmIndex =  unlist(indexProbes(newAB, 'pm'))
+     subIndex = match(tmp(probe.env.orig$x, probe.env.orig$y,
+                             cdf=cdfpackagename), pmIndex)
+
+     rm(newAB)
+     iNA = which(is.na(subIndex))
+
+     if(length(iNA)>0){
+         ipos = grep(probepackagename, search())
+         assign(probepackagename, probe.env.orig[-iNA,], pos=ipos)
+     }
+ }
```

Probeset level to gene level

It is worth noting that RMA yields probeset expression levels. However, there can be several probesets that map to a single gene in an Affymetrix array. If gene level expression is required (instead of probeset expression) the following code will average over probesets for each gene

```
> #Function to average the expression of
> #probesets which map to same gene
> probeset2genelevel = function(onesample)
+   return(tapply(onesample, factor(c_df$genename), mean))
> #Average for each column/array
> c_gene_data = apply(exprs(yeast.rma), 2, probeset2genelevel)
```

For the *S. cerevisiae* data, the majority of genes only have a single probeset. However, thirty five genes have two or three associated probesets. In this case study, we will study genes at the probeset level.

2 Genetic regulatory network inference

Recent research in the analysis of microarray experiments has produced several methods for determining plausible transcriptional regulation networks from the data; see, for example, [4–7]. Such networks can provide valuable insight into the underlying biological mechanisms producing the data. The networks typically consist of nodes (representing genes or proteins) and edges between nodes (representing relationships between genes). Methods also exist for inferring dynamic gene association networks in which the direction of causation is represented by an arrow. One such method (with an easy-to-use R package developed by the Strimmer lab) uses a shrinkage approach to calculate the partial correlation coefficient and works for both static and dynamic (time-course) data; see [8–10]. Their heuristic algorithm is fast and so provides a quick insight in the structure of the network. It works by first creating a `longitudinal` R object. In our illustration, we use data on the top one hundred differentially expressed genes. The data are stored in a matrix `m`, where the rows are genes and the fifthteen columns are the arrays. In order to use the R functions in their package, as we have time course data rearrange the row order according to the time points. The first three rows of the resulting matrix `mnew` are data on the three mutant arrays at time $t = 0$, the next three arrays at time point $t = 60$, and so on. This is achieved by using the commands

```
> exp_fac = with(exp_fac, exp_fac[order(strain, tps, replicates), ])
> #Construct a longitudinal object
> library(GeneNet)
> ngenes = 100
> m = yeast.matrix[ii[1:ngenes],]
> mnew = m[,exp_fac$data_order[1:15]]
> mlong = as.longitudinal(t(mnew), repeats=3, time=0:4)
```

Next the partial correlations are computed and then (local) values are assigned to all possible edges, from which the important edges can be determined according to a threshold criteria. Finally a Graphviz[1] file is outputted to generate the graph. Figure 1 shows the resulting network for these data and is generated by using

```
> #Compute partial correlations
> pcor.dyn = ggm.estimate.pcor(mlong, method = 'dynamic')
> #Assign (local) fdr values to all possible edges
> m.edges = network.test.edges(pcor.dyn, direct=TRUE)
> dim(m.edges)
> #Construct graph containing top edges
> m.net = extract.network(m.edges, method.ggm='number', cutoff.ggm=100)
> #Construct a Graphviz dot file
> rnames = vector('list', length(1))
> rnames = c_df$genename[ii[1:ngenes]]
> network.make.dot(filename='net.dot', m.net, rnames, main='Yeast Network')
```

[1]See http://www.graphviz.org/ for details.

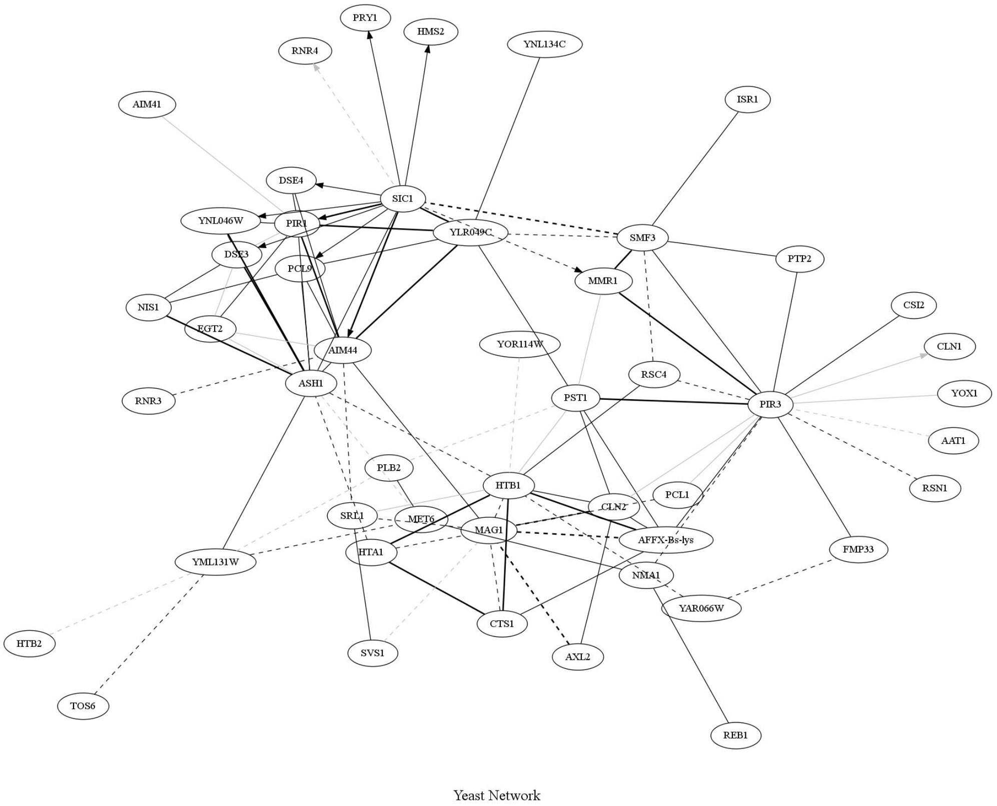

Figure 1: Gene network inferred from the yeast microarray data set. Black and grey indicate positive and negative (partial) correlation respectively.

Note that, in the function `ggm.estimate.pcor`, the default method `static` employs the function `pcor.shrink`, whereas the `dynamic` method uses `dyn.pcor`. The difference between the two estimators is that the latter takes the spacings between time points into account if the input consists of multiple time course data (which must be provided as a `longitudinal` object).

3 List of R packages

Package	Version	Package	Version
affy	1.24.2	IRanges	1.4.11
affyPLM	1.22.0	limma	3.2.1
AnnotationDbi	1.8.1	Mfuzz	2.4.0
ArrayExpress	1.6.1	timecourse	1.18.0
Biobase	2.6.1	yeast2probe	2.5.0
Biostrings	2.14.12	yeast2cdf	2.5.0
gcrma	2.18.1		

Table 1: List of packages from Bioconductor used in this paper

Package	Version	Package	Version
bitops	1.0-4.1	GeneNet	1.2.4
caTools	1.10	gplots	2.7.4
corpcor	1.5.5	grid	2.10.1
e1071	1.5-22	gtools	2.5.0
fdrtool	1.2.6	MASS	7.3-5
gdata	2.7.1	longitudinal	1.1.5

Table 2: List of packages from the CRAN repository used in this paper

4 Additional figures

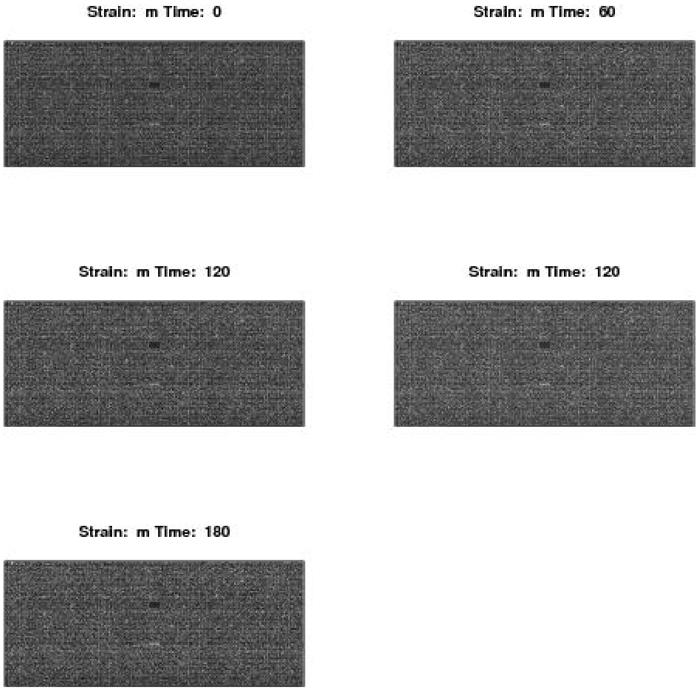

Figure 2: Image plots of the mismatch and perfect match probe intensities for the first replication of the mutant yeast strain. The corresponding times are indicated in the plot.

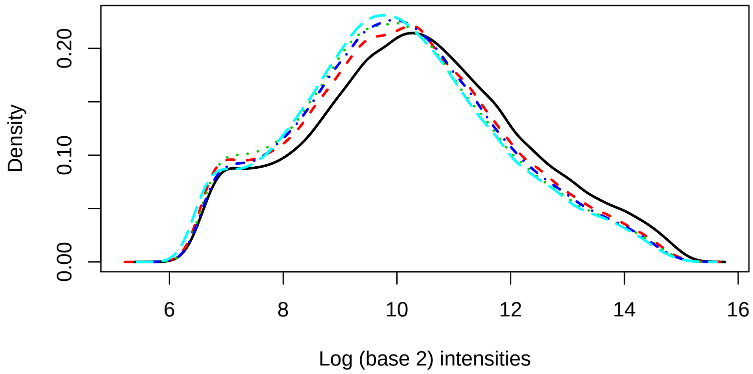

Figure 3: Density plots for the first replication of the mutant yeast strain.

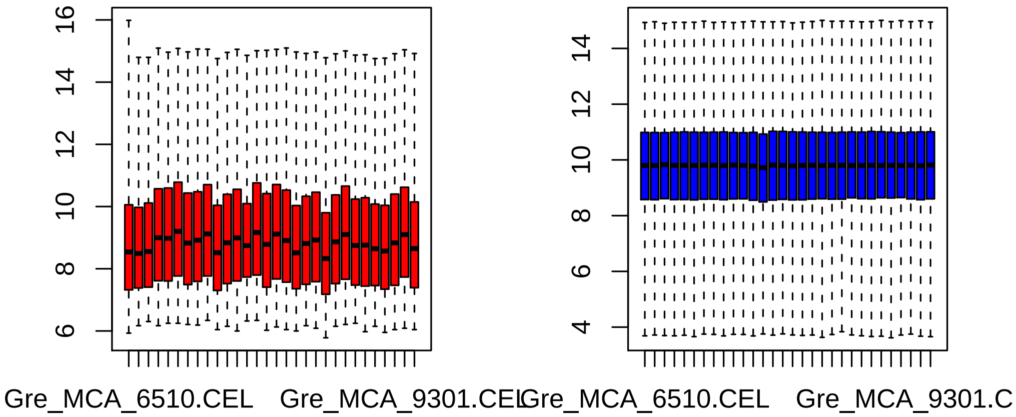

Figure 4: Boxplots of the raw and normalised intensities. The default boxplot is to include both PM and MM intensities, whereas for the density plots in Figure 3 the default is for only the PM intensities.

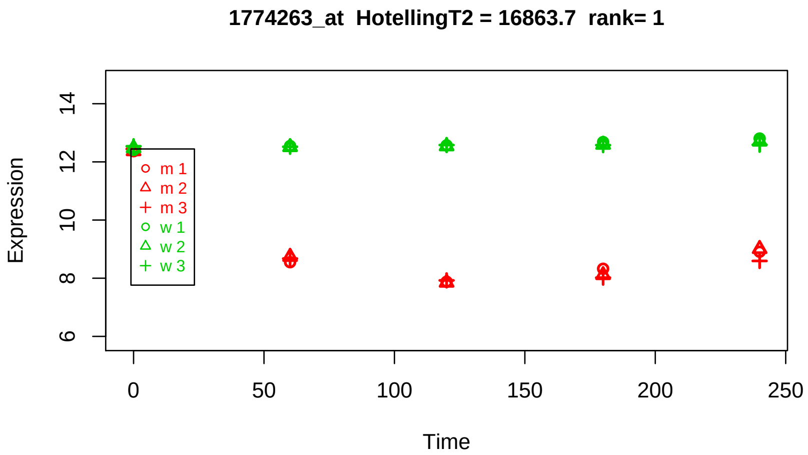

Figure 5: Time course expression levels for the top differentially expressed gene, ranked by their Hotelling statistic using the `timecourse` library.

References

1. **Pombe and Cerevisiae Filter** [http://www.affymetrix.com/Auth/support/downloads/mask_files/s_cerevisiae.zip].
2. **Yeast Annotation File** [http://www.affymetrix.com/Auth/analysis/downloads/na24/ivt/Yeast_2.na24.annot.csv.zip].
3. Gregory Alvord W, Roayaei JA, Quiñones OA, Schneider KT: **A microarray analysis for differential gene expression in the soybean genome using Bioconductor and R.** *Briefings in Bioinformatics* 2007, **8**:415–31, [http://www.ncbi.nlm.nih.gov/pubmed/17906332].
4. Dobra A: **Sparse graphical models for exploring gene expression data**. *Journal of Multivariate Analysis* 2004, **90**:196–212, [http://linkinghub.elsevier.com/retrieve/pii/S0047259X04000259].
5. Husmeier D: **Sensitivity and specificity of inferring genetic regulatory interactions from microarray experiments with dynamic Bayesian networks.** *Bioinformatics (Oxford, England)* 2003, **19**(17):2271–82, [http://www.ncbi.nlm.nih.gov/pubmed/14630656].
6. Yu J, Smith VA, Wang PP, Hartemink AJ, Jarvis ED: **Advances to Bayesian network inference for generating causal networks from observational biological data.** *Bioinformatics (Oxford, England)* 2004, **20**(18):3594–603, [http://www.ncbi.nlm.nih.gov/pubmed/15284094].
7. Lee I, Date SV, Adai AT, Marcotte EM: **A probabilistic functional network of yeast genes.** *Science (New York, N.Y.)* 2004, **306**:1555–8, [http://www.ncbi.nlm.nih.gov/pubmed/15567862].
8. Schäfer J, Strimmer K: **An empirical Bayes approach to inferring large-scale gene association networks.** *Bioinformatics (Oxford, England)* 2005, **21**:754–64, [http://www.ncbi.nlm.nih.gov/pubmed/15479708].
9. Opgen-Rhein R, Strimmer K: **Using regularized dynamic correlation to infer gene dependency networks from time-series microarray data**. In *Proceedings of the 4th International Workshop on Computational Systems Biology* 2006:73–76.
10. Opgen-Rhein R, Strimmer K: **From correlation to causation networks: a simple approximate learning algorithm and its application to high-dimensional plant gene expression data.** *BMC Systems Biology* 2007, **1**:37, [http://www.ncbi.nlm.nih.gov/pubmed/17683609].
